# An Unusual Case of Psoriatic Arthritis With Peliosis Hepatis

**DOI:** 10.7759/cureus.89363

**Published:** 2025-08-04

**Authors:** Boutaina Zerouali, Kawtar Nassar, Soukaina Zaher, Nisrine Bennani Guebessi, Saadia Janani

**Affiliations:** 1 Department of Rheumatology, Faculty of Medicine and Pharmacy, Ibn ROCHD University Hospital, Casablanca, MAR; 2 Department of Anatomical and Cellular Pathology, Faculty of Medicine and Pharmacy, Ibn ROCHD University Hospital, Casablanca, MAR

**Keywords:** il-17 inhibitor, peliosis hepatis, psoriatic arthritis, rheumatic diseases, tnf inhibitor

## Abstract

Peliosis hepatis is a rare condition characterized by dilation of the hepatic sinusoids and the presence of multiple blood-filled cystic spaces within the liver parenchyma. It has been associated with a variety of etiologies, including infectious diseases, immunological disorders, malignancy, and certain medications. We report a case of a 24-year-old male who presented with polyarthritis lasting two months. His medical history revealed a family and personal history of psoriasis, with no known drug use. The physical examination revealed psoriatic lesions localized to specific areas, accompanied by synovitis in his elbows, wrists, ankles, and knees. Biological tests showed elevated gamma-glutamyl transferase (298 IU/L, normal range: 12-64 IU/L) and total alkaline phosphatase (341 IU/L, normal range: 11-55 IU/L), while hepatitis A, B, and C serologies were negative. Additionally, a broad panel of autoimmune antibodies (including anti-nuclear, anti-Ro/SSA, anti-M2, anti-SP100, anti-SLA, and anti-LKM1) were all negative. The abdominal Doppler ultrasound and liver MRI with gadolinium contrast have shown homogeneous hepatosplenomegaly. A liver biopsy revealed features consistent with peliosis hepatis. Imaging studies revealed sacroiliac sclerosis, leading to a diagnosis of psoriatic arthritis complicated by peliosis hepatis. The patient initially received secukinumab (300 mg monthly), with partial improvement, followed by a switch to etanercept, which resulted in a significant clinical and biochemical response over six months. This case highlights the importance of considering peliosis hepatis in patients with psoriatic arthritis who present with liver enzyme abnormalities. It also demonstrates the safety and efficacy of anti-tumor necrosis factor (TNF) agents, particularly etanercept, in managing this condition compared to anti-IL17 therapies.

## Introduction

Peliosis hepatis is a rare hepatic condition characterized by dilated sinusoidal spaces filled with blood, often associated with a variety of etiologies, such as infections, autoimmune diseases, neoplasms, and drug toxicity [1]. The condition remains poorly understood in terms of its pathogenesis but has been recognized in the context of certain rheumatic diseases, including psoriatic arthritis [2]. The association between peliosis hepatis and chronic inflammatory diseases is extremely rare, making its recognition and management particularly challenging. Certain drugs, such as methotrexate and corticosteroids, may induce or exacerbate peliosis hepatis, complicating treatment choices for these patients. In this report, we present a case of psoriatic arthritis complicated by peliosis hepatis, emphasizing the clinical features, diagnosis, and treatment strategies.

## Case presentation

**Figure 1:**
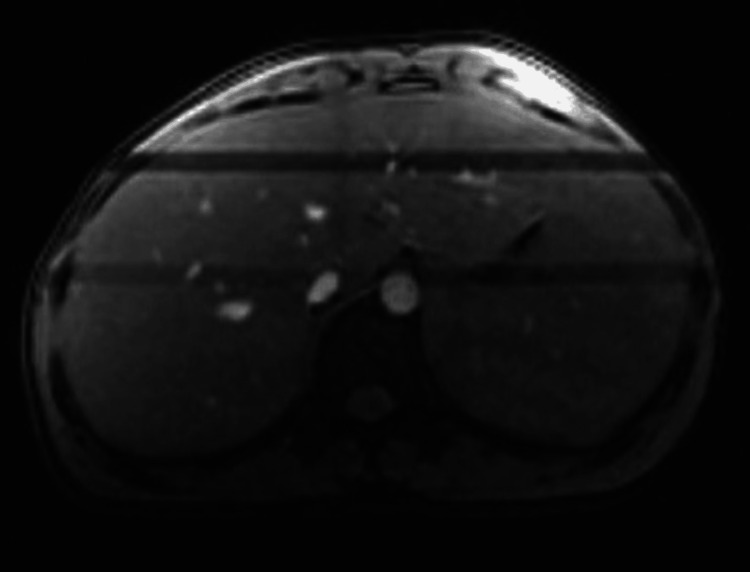
Abdominal MRI showed homogeneous hepatosplenomegaly.

A 24-year-old male patient with a history of psoriasis presented with a two-month history of polyarthritis. His medical background revealed no drug or alcohol use. Physical examination revealed localized psoriatic plaques, axillary lymphadenopathy, and synovitis in the elbows, wrists, ankles, and knees, in the context of unexplained weight loss. Laboratory parameters revealed elevated levels of gamma-glutamyl transferase (298 IU/L, normal range: 11-55 IU/L) and alkaline phosphatase (341 IU/L, normal range: 40-150 IU/L), while alanine aminotransferase, aspartate aminotransferase, serological tests for hepatitis A, B, and C, and HIV infection were negative. Additionally, a range of autoimmune antibodies, including anti-nuclear antibodies, anti-Ro/SSA, anti-M2, anti-SP100, anti-SLA, and anti-LKM1, were found to be negative. Moreover, the markers for latent tuberculosis screening were also negative. As part of the evaluation for unexplained weight loss, a cervico-thoraco-abdomino-pelvic CT scan was performed, revealing homogeneous hepatomegaly with no significantly enlarged lymph nodes. The abdominal Doppler ultrasound and liver MRI with gadolinium contrast have shown homogeneous hepatosplenomegaly without any associated abnormalities (Figure 1).

**Figure 2:**
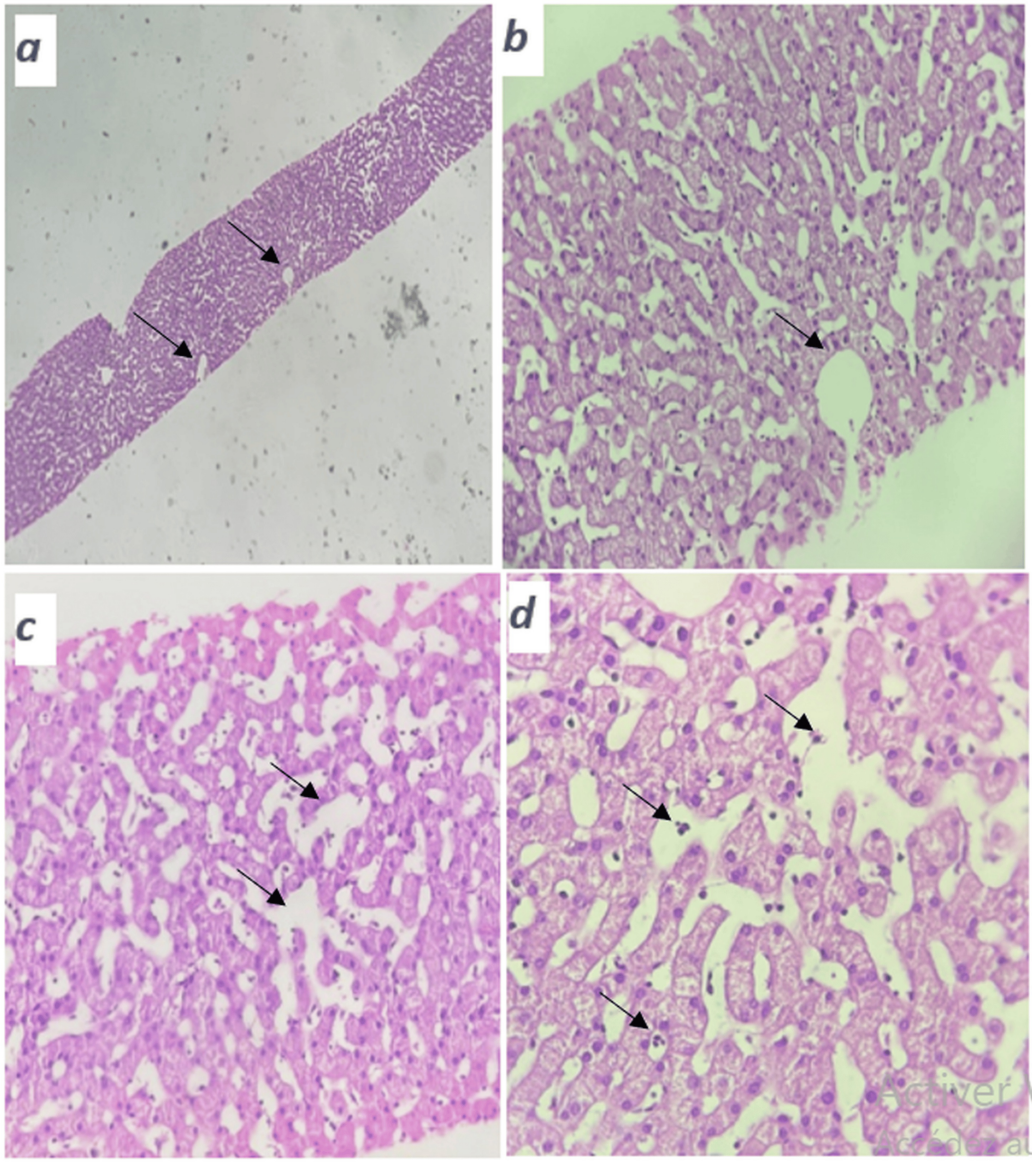
Histopathological findings of the liver biopsy showed sinusoidal dilatation and cholangiolar proliferation. Histopathological examination of the liver biopsy specimen showing dilated central vein and sinusoidal spaces (arrows) within the hepatic lobule (hematoxylin and eosin staining, original magnification ×100 {a}, ×200 {b}, and ×400 {c}). Cholangiolar proliferation (arrows) with inflammatory infiltrates in the portal spaces (hematoxylin and eosin staining, original magnification ×400 {d}).

**Figure 3:**
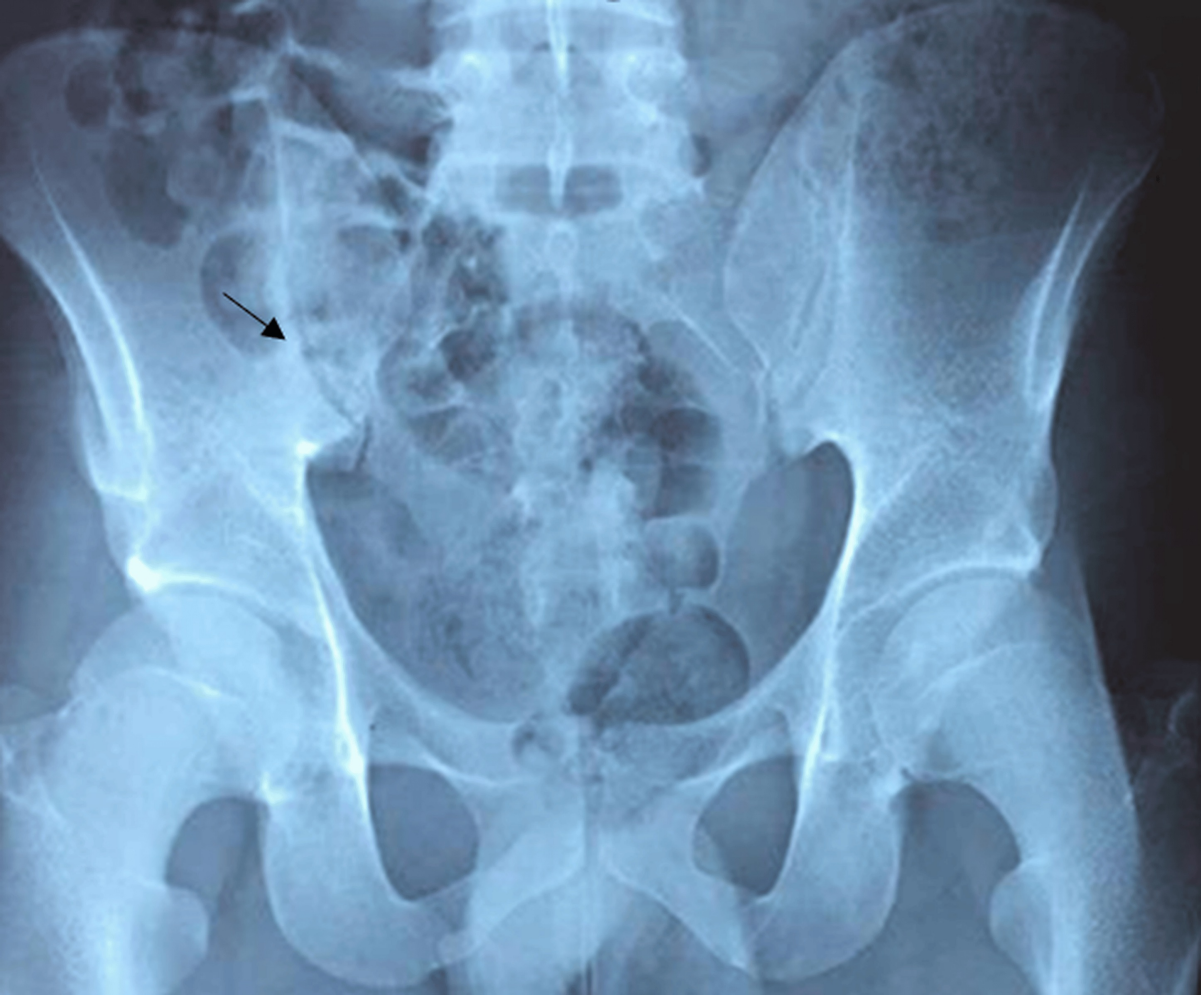
The pelvic radiograph showed condensation of sacroiliac joints. The arrow indicates subchondral sclerosis of the sacroiliac joints.

**Figure 4:**
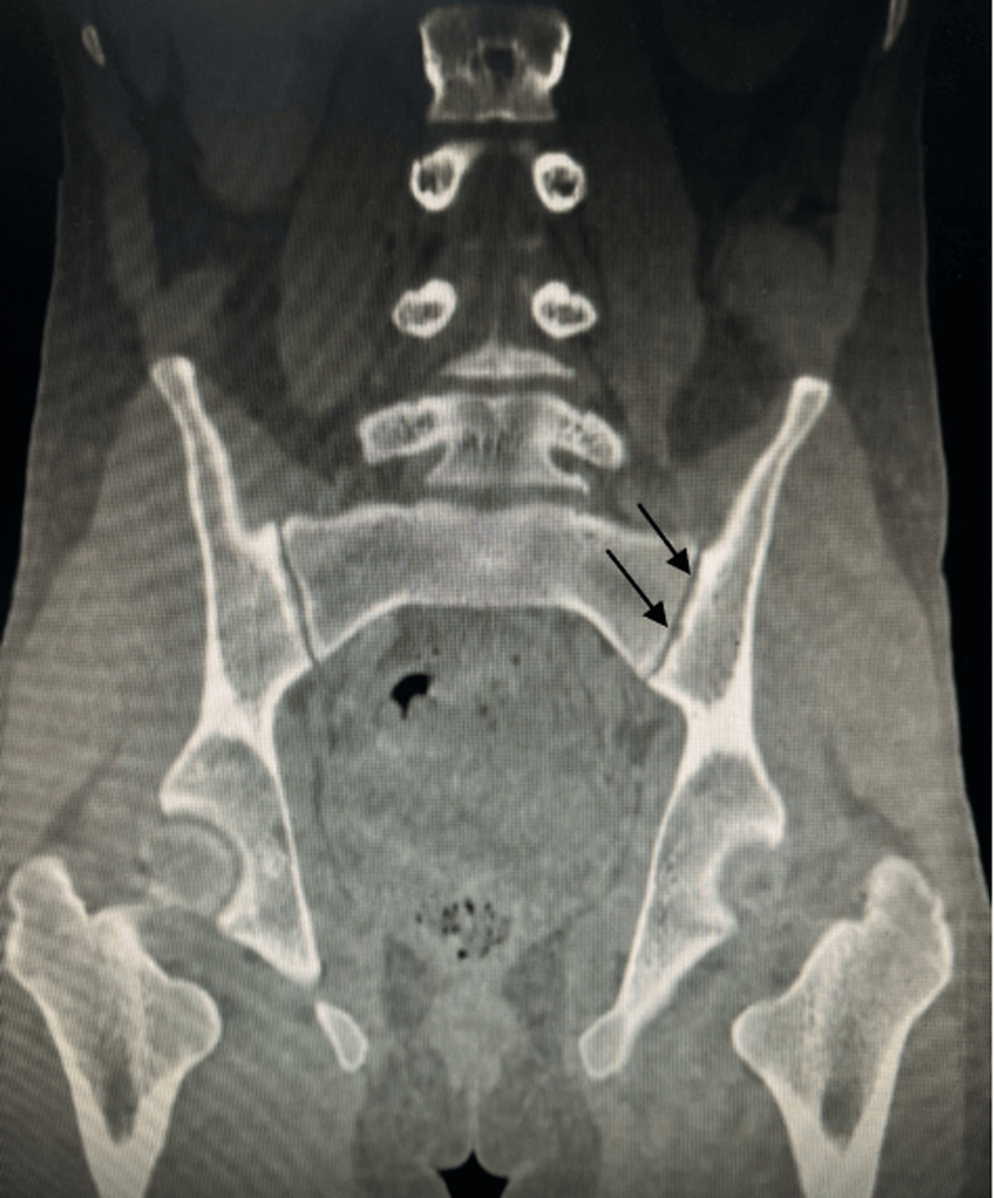
The CT scan of the sacroiliac joints showed sacroiliitis. The arrows highlight radiological signs of sacroiliitis, referring to subchondral sclerosis and erosions of the sacroiliac joints.

A liver biopsy revealed the characteristic features of peliosis hepatis, with no signs of hepatic steatosis, malignancy, or autoimmune hepatitis (Figures 2a-2d). Radiographs of the pelvis showed condensation of the iliac margins (Figure 3), which was also confirmed by the pelvic phase of the cervico-thoraco-abdomino-pelvic CT scan (Figure 4).

Since the patient was asymptomatic at the sacroiliac joints, an MRI was not performed. The diagnosis of psoriatic arthritis with peliosis hepatitis was established clinically and histologically. Additionally, the patient fulfilled the Classification Criteria for Psoriatic Arthritis (CASPAR) criteria for psoriatic arthritis [3]. The patient was initially treated with secukinumab, administered at weeks zero, one, two, three, and four, followed by monthly doses, resulting in improvement in liver enzyme levels. However, inflammatory markers remained elevated, and the patient continued to experience synovitis in both wrists. After three months, treatment was switched to etanercept (50 mg/week), resulting in a more significant clinical response, with no signs of synovitis and a biological improvement marked by a decrease in C-reactive protein to 7.3 mg/L. The Disease Activity in Psoriatic Arthritis (DAPSA) score decreased from 56 (indicating high disease activity) to 10 (low disease activity) [4].

## Discussion

We report a case of psoriatic arthritis associated with peliosis hepatis. A similar case has previously been reported in the literature [5]. Peliosis hepatis is a benign condition that primarily affects the liver but can also involve other organs like the spleen, kidneys, and lymph nodes [6]. While often asymptomatic and found incidentally [1], it can lead to serious complications, including hepatomegaly, portal hypertension, and even hepatic rupture in some cases [7]. The condition’s pathogenesis is not entirely understood, but it has been linked to drug use (e.g., methotrexate and corticosteroids) [8], infections (e.g., HIV and tuberculosis) [9,10], autoimmune diseases (e.g., lupus, dermatomyositis, or Takayasu arteritis) [11-14], and malignancies [15,16].

In the context of psoriatic arthritis, both inflammation and elevated levels of vascular endothelial growth factor may play a role in the development of peliosis hepatis. Psoriatic arthritis is associated with increased vascular changes due to the secretion of pro-inflammatory cytokines, such as tumor necrosis factor (TNF)-alpha and vascular endothelial growth factor, which may contribute to the development of peliosis hepatis [17]. Histopathologically, peliosis hepatis is characterized by dilated sinusoidal spaces and blood-filled cystic lesions [18]. Histopathological examination of the liver biopsy revealed these features, which can be confirmed by advanced imaging techniques, such as gadolinium-enhanced MRI [1].

In terms of management, treating the underlying cause of peliosis hepatis is essential. For our patient, the challenge was balancing the treatment of psoriatic arthritis with the potential exacerbation of liver dysfunction. Although methotrexate is a first-line treatment for psoriatic arthritis, its use in the presence of peliosis hepatis is controversial due to the risk of worsening liver involvement [19]. In contrast, biologic therapy, such as etanercept, a tumor necrosis factor inhibitor, proved effective in our case in improving both liver function and psoriatic arthritis symptoms. Interestingly, secukinumab, an IL-17 inhibitor, did not provide the same level of benefit.

The successful management of this case with etanercept highlights the potential for TNF inhibitors to safely treat psoriatic arthritis complicated by peliosis hepatis. It also underscores the importance of monitoring liver enzymes during treatment and considering alternative therapies when necessary.

## Conclusions

Peliosis hepatis should be considered as a potential cause of abnormal liver enzyme levels in patients with psoriatic arthritis. The association between these conditions is likely due to shared inflammatory pathways and increased vascular endothelial growth factor. Clinicians should be cautious when prescribing treatments, particularly methotrexate and glucocorticoids, in patients with psoriatic arthritis and peliosis hepatis. This case emphasizes the utility of TNF inhibitors like etanercept in managing this challenging association, providing better outcomes than IL-17 inhibitors. Further studies and long-term follow-up are needed to confirm these findings.
